# The Occurrence and Co-Occurrence of Harsh Parenting and Family Conflict in Hong Kong

**DOI:** 10.3390/ijerph192316199

**Published:** 2022-12-03

**Authors:** Qiqi Chen, Camilla Kin Ming Lo, Mengtong Chen, Ko Ling Chan, Patrick Ip

**Affiliations:** 1 Department of Social Work, School of Sociology and Anthropology, Xiamen University, Xiamen 361005, China; 2 Department of Applied Social Sciences, The Hong Kong Polytechnic University, Hung Hom, Hong Kong; 3 Department of Social Work, The Chinese University of Hong Kong, Sha Tin, Hong Kong; 4 Department of Paediatrics and Adolescent Medicine, LKS Faculty of Medicine, The University of Hong Kong, Pokfulam, Hong Kong

**Keywords:** harsh parenting, family conflict, co-occurrence, family satisfaction, Hong Kong

## Abstract

The violation of children’s right to a safe home environment is a major public health problem in need of serious attention. Evidence has been limited about the family characteristics that go with the co-occurrence of harsh parenting and family conflict. By using a representative community sample of Hong Kong families, this study aims to examine the prevalence and risk factors of harsh parenting and family conflict. This study was conducted using a secondary analysis obtained from the 2017 Hong Kong Family Survey with a sample size of 1926 respondents who have children. Results showed that participants’ ages are negatively related to the occurrence and co-occurrence of harsh parenting and/or family conflict. Married mothers reported less family conflict. Fathers with lower education levels reported more experiences of family violence. Mothers reporting a higher level of family satisfaction were less associated with harsh parenting. This study provides insights into the unique and shared familial elements that prevent harsh parenting and family conflict and help facilitate the development of effective intervention strategies for family violence co-occurrence. Family-based prevention for family violence may screen for the presence of harsh parenting and family conflict and take into consideration these signals to better support families in need.

## 1. Introduction

From the perspective of children, family violence encompasses their direct maltreatment and their witnessing of conflicts among family members [1]. Child maltreatment is a public health problem that brings enduring consequences for victims [2]. Much research has revealed the risk factors for child maltreatment: these include children of a younger age, children with special physical or educational needs, caregivers with addictive behaviors or mental health issues, harsh parenting, and a violent family or community environment [3,4,5]. Child maltreatment and witnessing family conflict were found to be highly prevalent in Hong Kong. For example, a study revealed that 13.1% of children experienced direct abuse, and 6.5% witnessed parental violence [6]. Research suggests that one type of violence within the family is likely to be a risk factor for other types of family violence [6]. The co-occurrence of two or more forms of family violence can lead to a range of adverse cognitive, emotional, and behavioral problems in affected children [1]. A study in Hong Kong revealed a prevalence rate of 12.3% for the co-occurrence of parental violence and child maltreatment [7]. Recent studies examining partner violence and child maltreatment found it attenuated in the same statistical model, which means that the occurrence of child maltreatment and partner violence has a greater effect on victims [8]. Children exposed to direct maltreatment and indirect family conflicts show more internal and external problems, including greater rates of depression, eating disorders, and academic and social problems [9,10,11]. Unpacking the specific and shared family characteristics engendered among family members may help reconcile these conflicting patterns of family violence [12].

Increasing attention has been paid to understanding common factors associated with harsh parenting and family conflict [13]. Research shows that individual factors such as being male, having psychopathology, and exhibiting addictive behaviors make one more likely to perpetrate partner violence [13]. In a study of families in 24 developing countries, 29% of parents reported believing that physical punishment is necessary to rear a child [14]. Studies found some parenting differences between fathers and mothers: for example, the negative effects of maternal control are especially evident in children under the age of five [15]. Family disadvantages such as poverty and increased financial insecurity may increase the family stress level and therefore lead to more harsh parenting [16,17]. Changes in daily routines (caused by things such as residential mobility or quarantine at home) may also increase the risk of family conflicts [18]. Economic hardship can exacerbate the challenges of parenting, causing an additional source of parental stress and, consequently, a greater risk of harsh parenting [19]. Extant research also demonstrates that parental stress is related to higher rates of family conflicts [20,21]. During the COVID-19 pandemic, school closures resulted in additional economic hardships and uncertainty for families, exposing children to higher levels of risk for abuse and neglect. Punishment of a child by teachers and childcare service providers has been prohibited by Hong Kong’s laws; however, parents’ use of punishment is endorsed by Chinese culture in Hong Kong [7]. Studies in Hong Kong have found that Chinese parents also believe the infliction of physical pain as punishment is necessary for training children to endure physical hardship [7,22].

Family systems theory claims that families are organized in subsystems such as parent–child, marital, and sibling subsystems, which may all have specific effects on child development [23]. Child dysfunction and maladjustment often develop in the context of poor parent–child relationships or distressed marriages [24]. Research has suggested that the co-occurrence of family violence can be understood as a within-individual phenomenon occurring across individuals or both [7]. For example, the co-occurrence of harsh parenting and parental violence could be that a husband abuses his wife and controls the child, that a husband abuses his wife and then the abused wife controls the child, or that a husband abuses his wife and then they both inflict harsh parenting on the child. This impacts more than family dynamics and the well-being of family members [25,26], as families with lower levels of parental supervision have an increased risk of children experiencing extra-familial victimization [27,28]. A study has shown that growing up in a violent family also increases the risk of children becoming perpetrators or victims of violence in intimate relationships at a later age [11]. A gender difference was found within this violence co-occurrence: an increased potential for victimized mothers’ harsh parenting was attributable to the abused women’s higher levels of distress and unsolved interpersonal difficulties [29]. Therefore, the victimization of mothers can lead to the victimization of children.

Family systems theory has also suggested that the impacts of subsystems on children are dynamic and multipath within the functioning of the whole family system [30]. For example, parents with higher marital satisfaction may more effectively manage their negative emotions and respond to children’s adverse behaviors, while parents who experience marital conflicts and dissatisfaction may be more irritable and rely on power-assertive techniques to manage children’s misbehaviors [23,31]. High marital satisfaction can be transmitted through family functioning; family functioning was positively predictive of a more harmonious family environment and higher life satisfaction for children [32]. On the other hand, empirical research in Hong Kong found that lower marital satisfaction is associated with harsh parenting, and harsh parenting plays a moderating role in the relationship between mothers’ marital satisfaction and children’s externalizing behavioral problems [33,34]. Authoritarian parenting and parental control were also the core family factors that were negatively related to children’s life satisfaction [23,35].

Harsh parenting has negative physical and mental health consequences for children, including individual anxiety, behavioral problems, aggression, social withdrawal, low self-esteem, and a higher economic burden for societies at large. Limited studies have compared the unique and shared roles of family characteristics in the context of harsh parenting and family conflict in Hong Kong. Based on earlier research [13,16], the following family factors were selected: socioeconomic status (which was indicated by parental education level and occupation); marital status of parents; and living status of the family. By using a representative sample from Hong Kong’s Family Surveys, the current study provides updated evidence on family characteristics of family conflict and harsh parenting. The study aims to explore the individual and family characteristics of harsh parenting and family conflict in Hong Kong families and compare the influence of gender on the family characteristics related to harsh parenting and family conflict.

## 2. Materials and Methods

### 2.1. Study Design and Procedure

This study used data from the family surveys carried out by the Family Council of the Home Affairs Bureau in Hong Kong. The surveys were commissioned on a biannual basis beginning in 2011 to provide updated information on changes and development among Hong Kong families. The surveys cover various themes, including family structure, family functioning, social support networks, parenthood, satisfaction with work and family life, and awareness of family-related programs. Based on the Hong Kong Census and Statistics Department Frame of Quarters, the surveys employed a 2-stage stratified sample design within the 6500 living quarters. In the first stage, we required respondents aged 15 or above and enumerated 3000 eligible living quarters. In the second stage, one individual was randomly selected from each household to complete the family survey. The selection method was based on the last birthday method. The average response rate to the surveys was 63.5%. An estimates level was adopted within ±2.2 percentage points at a 95% confidence level. The survey sampled all persons regardless of gender, sexual orientation, religion, geography, disability, language, and culture. The current study included a sample size of 1926 respondents from the 2017 family survey who were aged 15 or above and had children. This sample provided sufficient data for analysis on the topic. The study process was approved by the ethical committee of the Home Affairs Bureau of the Government of the Hong Kong Special Administrative Region.

### 2.2. Measures

#### 2.2.1. Harsh Parenting

Harsh parenting was measured with the subscale of the Chinese Family Assessment Instrument (CFAI) [36]. The CFAI is a 33-item measure classified into five dimensions to assess family functioning: mutuality, communication and cohesiveness, conflict and harmony, parental concern, and parental control. The parental control subscale consists of three items encompassing parental control behaviors towards children, including “Parents scold and beat children”, “Parents force children to do things”, and “Parental control is too harsh”. Respondents were asked to assess their behaviors on a 5-point Likert scale (ranging from 1 = “Does not fit our family” to 5 = “Very much fits our family”). A higher score indicates more parental control behaviors towards children. We referred to recent research, which suggested a cutoff point of happened “at least once” to compare the violent experiences [37]. The Cronbach reliability statistics for this scale were satisfactory (α = 0.7).

#### 2.2.2. Family Conflict

The CFAI conflict and harmony subscale was adopted to assess family conflict. It consists of six questions such as “No mutual concern”, “Much friction among family members”, “Frequent fighting among family members”, “Not much quarrelling among family members”, “Lack of harmony among family members”, and “Poor marital relationship between parents”. Ratings were also expressed on a Likert scale of 5, with a score of 1 denoting “Does not fit our family” and one of 5 denoting “Very much fits our family”. A higher score implied that the family had more conflicts, such as fights and quarrels. The Cronbach reliability statistics for this scale were satisfactory (α = 0.7).

#### 2.2.3. Family Satisfaction

Family satisfaction was assessed with a single item on a Likert scale ranging from 1 (“Very dissatisfied”) to 5 (“Very satisfied”). A higher score indicated the participant’s greater satisfaction with family life.

#### 2.2.4. Demographic Characteristics

Participants were asked to provide their demographic information, including age, gender, marital status, education level, occupation, and family living status. Education level was coded on a 3-point scale (0 = “Primary or lower education”; 1 = “Post-secondary education or above”; 2 = “Secondary education”). Marital status was coded as 0 = “Not married” (including divorced or separated, never married, and widowed) or 1 = “Married or cohabiting with a partner”. We referred to the classification by the Census and Statistics Department of Hong Kong and categorized the economic levels of the participants into four groups, including “economically inactive” (those retired, home-makers or students), “skilled, craft, workers, and elementary occupations”, “clerk, service/shop sales workers”, and “managers, professionals/associate professionals”. Living status was measured by whether the respondent rented living space or owned it.

### 2.3. Statistical Analysis

Descriptive analyses were first conducted to summarize the prevalence percentages of participants’ demographic characteristics. All the demographic and predictor variables were compared to participants’ experiences of harsh parenting and family conflict. We then conducted a series of multinomial logistic regression analyses to estimate the associations among demographic and family characteristics, satisfaction towards family, harsh parenting, and family conflict. We recorded and grouped those participants having had experiences of family violence into one of four clusters: “Some experience of family violence (either harsh parenting or family conflict)”, “Experience of harsh parenting only”, “Experience of family conflict only”, and “Experience of both harsh parenting and family conflict co-occurrence”. Those who reported both two types of harsh parenting and family conflicts were grouped as “Harsh parenting and family conflict co-occurrence”. All the predictor variables were compared between fathers and mothers to compare the impact of gender differences in predictor variables. Demographic characteristics and family satisfaction were computed as independent variables, and the occurrence and co-occurrence of harsh parenting and family conflict were adopted as dependent variables. All the above statistical analyses were completed using SPSS 26.0. The significance level was determined by two-tailed tests with a *p*-value of less than 0.05.

## 3. Results

### 3.1. Demographic Characteristics of the Participants

Table 1 demonstrates the demographic characteristics of the respondents in this study. The total sample was comprised of 1926 participants having children that completed the survey, of whom 452 (23.5%) reported harshly parenting their children; 986 (51.2%) reported family conflict, and 283 (14.7%) reported both harsh parenting and family conflict experiences. A majority of the participants (62.3%) were aged 55 or above, and 6.1% of them were between the ages of 15 and 34. A total of 72.1% of the overall sample were married or cohabitating with a partner. Regarding education level by gender, 10.6% of the fathers and 8.3% of the mothers completed secondary education or above. In terms of occupation, most of the fathers (52.4%) and mothers (69.0%) who responded were economically inactive. Although 30.6% of the fathers were skilled workers and 10.6% were professionals, only 14.5% and 3.3% of the mothers reported having these occupational statuses. Regarding housing status, 35.6% of the participants were tenants during the survey period, and 14.2% were homeowners. Differences were observed in some individual and family parameters across victimization types (Table 1). Around half of those in all age groups reported family conflicts, while only 16.0% among those aged 55 or above reported engaging in harsh parenting. One in four of those aged 15–34 and 35–54 reported violence co-occurrence, while only 9.6% of those aged 55 or above reported this.

### 3.2. Individual Characteristics of Harsh Parenting and Family Conflict

In order to explore the differences in predictive variables across violence types, we conducted a series of regression analyses by violence types, as shown in Table 2. In the analysis, each variable was computed with controls for other variables. Results showed that both harsh parenting (ORs ranged from 0.961 to 0.967, *p*s < 0.001) and family conflict (ORs ranged from 0.968 to 0.984, *p*s < 0.001) and their co-occurrence (ORs ranged from 0.957 to 0.966, *p*s < 0.001) were negatively related to participants’ ages, which means that younger respondents reported more experiences of violence in their lifetimes. This is applicable to both genders. Fathers (OR = 1.810, *p* < 0.05) and mothers (OR = 1.551, *p* < 0.01) who were in marital or cohabiting relationships reported significantly more cases of harsh parenting than those who were not married. Married mothers reported fewer family conflicts (OR = 0.605, *p* < 0.001), and married fathers reported more violence co-occurrence (OR = 1.963, *p* < 0.01) compared to those who were not married, while no significant difference was found among their sexual counterparts on these items. Fathers who reached an education level of lower secondary or below reported significantly more of the single type of violence experiences (OR = 1.980, *p* < 0.01) compared to those with higher-education degrees. No significant difference was found in the relationship between violence and mothers’ education or in the occupations of both genders.

### 3.3. Family Characteristics of Harsh Parenting and Family Conflict

By gender, we also compared the relationship between family satisfaction and violence experiences (Table 2). A higher level of family satisfaction was also negatively related to family conflict both for fathers (OR = 0.349, *p* < 0.001) and mothers (OR = 0.402, *p* < 0.001). Mothers’ reporting of family satisfaction was negatively related to the co-occurrence of harsh parenting and family conflicts (OR = 0.729, *p* < 0.05). No significant relationship was found between family satisfaction and harsh parenting.

## 4. Discussion

This study examined the prevalence of harsh parenting and family conflict in Chinese families from Hong Kong and explored the shared and unique risk factors of their occurrence and co-occurrence. In summary, the results indicated that participants’ ages were related to both harsh parenting and family conflict. Fathers with a lower level of education reported significantly more family violence, while married fathers reported more family violence co-occurrence.

Our findings showed that both harsh parenting and family conflict were negatively related to participants’ ages. This is consistent with the finding in previous studies that younger parents report more family violence experiences [12,38]. Younger parents may have limited parenting skills and experiences to help deal with the problematic behaviors of children or conflicts with parents [22]. Young couples with poor marital relationships can see this spill over into their parent–child interactions in ways such as harsh parenting and control over the child [7]. Exposure to conflict within the family context has an adverse impact on a young child [11]. Impaired social interactions within the family, such as conflict and violence, could also become a training ground for problem relationships outside the family [39]. Additionally, younger parents may experience more social and economic disruption, which can lead to parental stress and therefore increase the risk of harsh parenting [40,41]. Programs working with families should consider promoting stress-coping strategies and positive parenting to help younger couples mitigate the negative consequences of family and child-rearing stress.

We found that married mothers reported fewer family conflict experiences. Previous studies have also revealed that children living in a single-parent family reported more harsh parenting and witnessing parental conflict [11]. Mothers often play the role of the major caregiver and spend more time with their children than fathers [23]. Children from single-mother households may also exhibit more externally delinquent behaviors due to high levels of stress in the home and the inability of the single mother to solve these problems on her own [19]. This gender difference could be explained by the fact that married mothers shoulder more childcare and housework, which could reduce their marital satisfaction [23]. These findings shed light on future methods of preventing harsh parenting, which could include ensuring that caregivers (especially single mothers) are better supported and connected. This could be accomplished by including more family-based therapies that promote the mental health of the mothers instead of treating children in isolation [42]. Policies should prioritize resource allocation to single mothers by (for example) expanding access to counseling services that promote healthy coping strategies and improved communication in households where the parent is unmarried.

Results showed that fathers who were married and had lower education attainment reported more family violence. This is consistent with previous studies in Hong Kong and other Asian countries that show low parental educational attainment was associated with more harsh parenting [43,44]. In a traditional Chinese values system, harsh parenting and parental control are regarded as a form of loving children rather than showing hostility towards them [45,46]. Parents (especially fathers with lower educational attainment) tended to favor putting pressure on children so that they reach high levels of academic achievement and gain honor for the family. This attitude is common in Asian families [47,48]. However, this belief has been impacted by the Western cultural attitude, especially prevalent in Hong Kong, that harsh parenting is a violation of individual rights [23]. Recent studies have shown that emotional abuse of children was related to parents’ higher education level who would expect children’s better academic performance [49]. Further research is needed to compare these effects of socioeconomic and sociocultural stressors on parents of different educational attainments and to differentiate the mediation pathways in how families are affected by various stressors.

Regarding the roles of family satisfaction, we found that mothers who reported higher levels of family satisfaction were less likely to maltreat their children and that fathers’ reporting of family functioning was negatively related to family conflict. Family satisfaction was found to be associated with child development via the parent–child system in both China and the West, which supports the cross-cultural universality of the family system theory [23]. For example, harsh parenting was reported to play a mediating role between family satisfaction and children’s negative emotional statuses [50], and family functioning apparently plays a mediating role between child maltreatment experiences and children’s antisocial behaviors [11,51]. Changes in family dynamics and functioning—such as disruptions to social networks, family routines, and family resilience—may lead to increased family conflict and violence [52]. Higher levels of family conflict were associated with more serious mental health problems in family members [19]. These findings are extremely important during the time of COVID-19, particularly for those who are experiencing financial hardship [21]. In caregivers, negative emotional states due to limited resources and impaired family satisfaction during the crisis can manifest as negative behavioral patterns with those family members for whom they are caring [42]. Future research and practice regarding affected families should focus more on the potential increase in harsh parenting or family conflict prevention during crises.

The findings of this study should be interpreted with the following limitations. First, since the data employed for cross-sectional analysis were from a large family survey in Hong Kong, any potential causal inferences about the relations between risk factors and family violence could not be explored at this stage. Since temporality is a key component of causality, to better explore the mechanisms of social and cultural factors on characteristics of family violence co-occurrence, longitudinal designs are suggested in future research for studying the same group of participants. Second, the participants were primarily recruited from working families in Hong Kong and may not be representative enough for a cross-cultural comparison of people around the globe. In order to assess family violence more comprehensively, future research would benefit from recruiting families with different socioeconomic backgrounds. Moreover, previous studies suggested increased odds of some psychiatric and conduct disorders were associated with disrupted life routines and lower socioeconomic status [53]. This should be strengthened by trained community health workers targeted at disadvantaged groups such as at-risk individuals and families against the heightened risk of poor mental health. Third, we investigated a broad area of family conflict rather than detailed incidents. The self-report on within-family violence was subject to a social desirability bias that could lead to underreporting. To provide multidimensional intervention measures, an important consideration for future studies would be including more participants and taking into account more types of family violence.

A considerable body of literature has reported on the relationship between family hardship and harsh parenting [54,55], while there have been few investigations that made comparisons with other forms of family violence. Reducing single-mother stress could enable parents to adopt more appropriate parenting skills than parental control and harsh discipline. A majority of the existing child welfare systems and programs focus on separately training parents and have not resulted in the placement of children exposed to family conflict [56], while our findings promote a family-based approach that addresses the interconnectedness of the problems [19,57]. A more integrated approach to violence screening could be more effective in identifying neglected victims or victimization in the same family [13]. Future intervention programs for child protection and family violence prevention would benefit from focusing on improving family satisfaction for both fathers and mothers. The COVID-19 pandemic has brought substantial hardship and stress to families across the globe [58]. Many parents have had to cope with extra economical and emotional strains while being isolated from extra-familial support. These difficulties may further increase the risks of abuse and conflict across time [59]. Family therapies could be an effective way to reduce the negative effects of the parenting stress triggered by daily life. Recent research has demonstrated that systematic family therapy and evidence-based parenting interventions are more effective in reducing problematic child behaviors and maltreatment than individual-based therapies [60]. Therapists may also screen for the presence of parental stress in parent–child relationships and take into consideration the interrelated dynamics of these signals to support families in need.

## 5. Conclusions

Harsh parenting and family conflict are major social and public health problems, the characteristics of which can vary with changes in social development. Prevention of family violence would benefit from an estimation of risk and protective factors to address the problem and facilitate the development of effective intervention strategies. The present study contributes an important supplement to the literature by comparing the occurrence and co-occurrence of harsh parenting and family conflict in Hong Kong. It also shows significant correlations between family satisfaction and family violence co-occurrence. These findings may help foster family resilience and inform policy and practice to better support the needs of caregivers.

## Figures and Tables

**Table 1:** Prevalence of harsh parenting and family conflict by demographic characteristics.

N (%)	Total (*n* = 1926)	Some Experience of Family Violence (*n* = 1155)	Experience of Harsh Parenting Only (*n* = 452)	Experience of Family Conflict Only (*n* = 986)	Experience of Harsh Parenting and Family Conflict Co-Occurrence (*n* = 283)
Age					
15–34	6.1%	63.5%	38.6%	50.0%	24.8%
35–54	31.7%	70.5%	36.4%	57.5%	23.5%
55 or above	62.3%	55.9%	16.0%	49.4%	9.6%
Parents’ marital status					
Married	72.1%	60.2%	25.9%	49.6%	15.4%
Not married (never married, cohabiting, divorced, separated, widowed)	27.9%	62.8%	18.1%	57.9%	13.3%
Father’s education level					
Lower secondary or below	60.9%	63.1%	19.6%	55.0%	11.6%
Upper secondary	28.5%	60.6%	22.7%	51.0%	13.2%
Post-secondary or above	10.6%	54.7%	27.6%	44.0%	17.1%
Mother’s education level					
Lower secondary or below	67.3%	59.5%	22.0%	52.2%	14.9%
Upper secondary	24.5%	65.2%	33.0%	52.4%	20.1%
Post-secondary or above	8.3%	57.6%	29.3%	44.4%	16.2%
Father’s occupation					
Economically inactive	52.4%	58.0%	16.0%	52.5%	10.6%
Skilled, craft workers, and elementary occupations	30.6%	68.6%	24.7%	56.6%	12.7%
Clerk, service/shop sales workers	6.4%	65.2%	30.4%	54.3%	19.6%
Managers, professionals/associate professionals	10.6%	56.0%	32.9%	41.3%	18.4%
Mother’s occupation					
Economically inactive	69.0%	59.3%	25.4%	50.0%	16.3%
Skilled, craft workers and elementary occupations	14.5%	63.6%	20.2%	59.3%	15.7%
Clerk, service/shop sales workers	13.1%	64.9%	27.5%	51.6%	14.3%
Managers, professionals/associate professionals	3.3%	61.5%	37.5%	48.7%	25.0%
Living status					
Rent/shared rent	35.6%	64.0%	24.8%	56.0%	16.8%
Self-owned	14.2%	54.7%	21.3%	44.4%	11.4%

**Table 2:** The occurrence and co-occurrence of harsh parenting and family conflict.

OR [95% CI]	Harsh Parenting or Family Conflict				Harsh Parenting Only			
	Father		Mother		Father		Mother	
Age	0.972 *** [0.957, 0.986]	<0.001	0.965 *** [0.955, 0.975]	<0.001	0.961 *** [0.945, 0.977]	<0.001	0.967 *** [0.957, 0.977]	<0.001
Marital status								
Married	1.591 [0.993, 2.550]	0.054	0.797 [0.595, 1.068]	0.129	1.810 * [1.030, 3.182]	0.039	1.551 ** [1.126, 2.136]	0.007
Not married	1		1		1		1	
Education level								
Lower secondary or below	1.980 * [1.037, 3.782]	0.039	1.425 [0.823, 2.466]	0.206	1.332 [0.652, 2.721]	0.431	1.189 [0.679, 2.083]	0.545
Upper secondary	1.469 [0.776, 2.781]	0.237	1.470 [0.841, 2.570]	0.176	0.945 [0.474, 1.883]	0.872	1.492 [0.854, 2.609]	0.160
Post-secondary or above	1		1		1		1	
Occupation								
Economically inactive	0.840 [0.430, 1.640]	0.609	1.252 [0.553, 2.832]	0.590	0.720 [0.355, 1.462]	0.364	0.907 [0.428, 1.925]	0.800
Skilled, craft, workers, and elementary occupations	0.881 [0.460, 1.685]	0.701	0.965 [0.405, 2.302]	0.936	0.659 [0.335, 1.295]	0.226	0.478 [0.209, 1.094]	0.081
Clerk, service/shop sales workers	0.900 [0.387, 2.093]	0.806	1.015 [0.432, 2.383]	0.973	0.957 [0.410, 2.234]	0.920	0.586 [0.263, 1.306]	0.191
Managers, professionals/associate professionals	1		1		1		1	
Satisfaction with family	0.349 *** [0.246, 0.496]	<0.001	0.402 *** [0.308, 0.525]	<0.001	1.073 [0.755, 1.527]	0.694	0.852 [0.666, 1.090]	0.203
Cox and Snell R Square	0.161		0.178		0.060		0.072	
Nagelkerke R Square	0.218		0.240		0.092		0.107	
N	710		1155		709		1162	

Note. * *p* < 0.05, ** *p* < 0.01, *** *p* < 0.001.

## Data Availability

Data are available on request from the authors.
